# Interobserver Variability and Histopathologic Correlation of Lung Ultrasonography in a Bleomycin-Induced Mouse Model of Systemic Sclerosis

**DOI:** 10.3390/biomedicines14040738

**Published:** 2026-03-24

**Authors:** Göksel Tuzcu, Gökhan Sargın, Bilge Yılmaz, Yaşar Barış Turgut, Yiğit Uyanıkgil

**Affiliations:** 1 Department of Radiology, Faculty of Medicine, Aydın Adnan Menderes University, 09100 Aydın, Turkey; 2 Department of Rheumatology, Faculty of Medicine, Aydın Adnan Menderes University, 09100 Aydın, Turkey; gokhan_sargin@hotmail.com; 3 Department of Histology and Embryology, Faculty of Medicine, Ege University, 35100 İzmir, Turkey; blge98ylmz@gmail.com (B.Y.); yigituyanikgil@gmail.com (Y.U.); 4 Department of Nephrology, Faculty of Medicine, Aydın Adnan Menderes University, 09100 Aydın, Turkey; barroturgut@hotmail.com; 5 Department of Stem Cell, Health Science Institute, Ege University, 35100 İzmir, Turkey

**Keywords:** Ashcroft score, bleomycin, interobserver variability, interstitial lung disease, lung ultrasonography, pulmonary fibrosis, systemic sclerosis

## Abstract

**Objectives:** Interstitial lung disease (ILD) is a major cause of morbidity and mortality in patients with systemic sclerosis (SSc). This study aimed to evaluate interobserver variability and the relationship between lung ultrasonography (LUS) findings and histological fibrosis severity in a bleomycin (BLM)-induced mouse model of SSc. **Materials and Methods:** Twenty female BALB/c mice were randomly assigned to a control group (*n* = 10) or a BLM-treated group (*n* = 10). Pulmonary fibrosis was induced by daily subcutaneous administration of BLM for three weeks. Two blinded observers (a radiologist and a rheumatologist) performed LUS using a high-frequency linear probe and calculated scores based on B-line distribution. Lung fibrosis was evaluated by Masson’s trichrome staining and quantified using the Ashcroft scoring system. Interobserver agreement was assessed with Cohen’s kappa, and correlations were analyzed using Spearman’s rank test. **Results**: Control mice exhibited normal lung architecture, whereas all BLM-treated mice developed moderate to severe fibrosis, with significantly higher Ashcroft scores. LUS revealed multiple B-lines, pleural irregularities, and loss of A-lines in BLM-treated mice. LUS scores were considerably higher in the BLM group (*p* < 0.001). Radiologist-assessed scores showed a strong correlation with Ashcroft scores (ρ = 0.78), while rheumatologist-assessed scores demonstrated a moderate correlation (ρ ≈ 0.62). Interobserver agreement was moderate, with discrepancies mainly in intermediate fibrosis stages. **Conclusions:** LUS is a useful non-invasive method for semiquantitative assessment of pulmonary fibrosis in this SSc model. Its correlation with histological severity supports clinical relevance, while moderate interobserver variability highlights the need for standardized protocols and training.

## 1. Introduction

Systemic sclerosis (SSc) is a chronic autoimmune connective tissue disease marked by vasculopathy, immune dysregulation, and progressive fibrosis of the skin and internal organs, particularly the lungs [1]. Among all organ manifestations, interstitial lung disease (ILD) is a leading cause of morbidity and mortality in SSc, affecting up to 80% of patients during the disease course and significantly limiting long-term survival [2,3]. The clinical course of SSc-ILD varies from stable disease to rapid progression, underscoring the need for early detection and timely intervention [4].

Pulmonary fibrosis in SSc-ILD is characterized by excessive accumulation of extracellular matrix components, resulting in irreversible architectural distortion and impaired gas exchange [5]. High-resolution computed tomography (HRCT) remains the gold standard for ILD diagnosis, but its routine use is limited by cost, radiation exposure, and feasibility in longitudinal animal studies or resource-limited settings [6,7].

In recent years, lung ultrasonography (LUS) has emerged as a promising, non-invasive, radiation-free imaging modality for detecting ILD. LUS can identify key features such as B-lines, pleural irregularities, and subpleural consolidations, which reflect interstitial involvement [8,9]. However, in preclinical animal models, lung ultrasound is inherently operator-dependent, and differences in acquisition settings, probe angulation, and interpretation criteria may introduce variability, potentially limiting reproducibility and translational application [10,11].

The bleomycin (BLM)-induced murine model of pulmonary fibrosis closely mimics many histopathological and radiological features of human SSc-ILD and remains widely used for preclinical evaluation of novel diagnostic tools [12,13]. Yet the consistency of LUS-based detection in this model has not been systematically assessed, particularly with respect to interobserver variability.

This study aimed to evaluate the diagnostic agreement between two independent observers using LUS for detecting ILD in a mouse model of BLM-induced SSc.

## 2. Materials and Methods

### 2.1. Study Design and Ethical Approval

This controlled, randomized experimental study included 20 female BALB/c mice aged 6–8 weeks and weighing 18–22 g. Animals were housed under standard laboratory conditions (22 ± 2 °C, 12:12 h light–dark cycle) with ad libitum access to food and water. All animal procedures were approved by the Aydın Adnan Menderes University Animal Care and Use Committee (Approval No: 64583101/2025/006) and conducted in accordance with the NIH Guide for the Care and Use of Laboratory Animals.

### 2.2. Experimental Groups and Randomization

Mice were randomly assigned to two groups (*n* = 10 per group) using a computer-generated randomization list: Group 1 (Control): Received subcutaneous (s.c.) injections of 0.3 mL saline every other day for 21 days. Group 2 (BLM): Received s.c. injections of BLM (1 mg/kg in 0.3 mL) every other day into the shaved dorsal skin for 21 days. No animals were excluded. Sample size was determined a priori based on ethical considerations and the 3R principles, and to provide an exploratory estimate of interobserver agreement and histology–ultrasound correlation in a well-established BLM fibrosis model.

### 2.3. Lung Ultrasound Protocol

On day 21 following BLM administration, LUS was performed under light general anesthesia induced by intraperitoneal ketamine (100 mg/kg) and xylazine (20 mg/kg). Adequate anesthesia depth was confirmed by the absence of pedal withdrawal reflex while maintaining spontaneous respiration. Body temperature was maintained using a heating pad throughout the procedure.

Chest hair was removed with a depilatory cream to minimize acoustic artefacts. Imaging was conducted using a high-frequency linear probe (LA2–14A) connected to a Samsung RS80A ultrasound system (Samsung Medison, Seoul, Republic of Korea).

Each mouse was positioned in the supine position and scanned bilaterally by two independent observers (a radiologist and a rheumatologist, each with approximately 10 years of ultrasound experience). Both observers were blinded to the experimental group allocation and performed the examinations independently using the same predefined scanning protocol and imaging parameters. Ultrasound cine-loops were recorded during image acquisition and were subsequently analyzed offline. Each observer acquired and interpreted their own ultrasound images independently, and no information regarding the other observer’s findings was available during the examination or scoring process.

The thorax was divided into six standardized scanning zones per mouse, including upper and lower anterior, lateral, and posterior regions of both hemithoraces. A minimum of intercostal spaces 2–6 was examined bilaterally. Imaging parameters were standardized across all examinations: depth 2.5 cm, gain 50%, focal zone placed at the pleural line, and cine-loops of 3–5 s acquired at end-expiration.

B-lines were defined according to international consensus criteria as vertical, hyperechoic artifacts arising from the pleural line and extending to the bottom of the screen without fading. In each scanning zone, B-lines were scored semi-quantitatively on a scale from 0 to 3 based on their number and degree of confluence. The total LUS score for each animal was calculated by summing the regional scores. In addition, the presence or absence of A-lines and B-lines per hemithorax was recorded. This semiquantitative scoring approach has been previously applied in both clinical and experimental models of ILD [10].

The LUS examination protocol used in this study was derived from internationally accepted recommendations for lung ultrasound imaging and from previously published studies investigating ILD. In particular, the definition of B-lines and the semiquantitative scoring approach were based on consensus criteria proposed for lung ultrasound examinations [10].

Interobserver variability between the two observers was assessed using Cohen’s kappa statistic. Intraobserver variability was not evaluated because each observer performed a single independent assessment of the ultrasound images.

### 2.4. Histopathological Evaluation and Ashcroft Scoring

Following LUS examination, animals were euthanized and lung tissues were harvested. Specimens were fixed in 10% neutral-buffered formalin, embedded in paraffin, and sectioned at a thickness of 5 μm. Lung sections were stained with hematoxylin and eosin (H&E) and Masson’s trichrome for histological evaluation.

Pulmonary fibrosis severity was assessed using the Ashcroft scoring system [14,15], which grades fibrosis on a scale from 0 (normal lung architecture) to 8 (total fibrotic obliteration). For each lung, a minimum of 10 randomly selected, non-overlapping microscopic fields at ×200 magnification were evaluated. Scoring was performed independently by two blinded experts (a pathologist and a pulmonologist), and the final Ashcroft score for each animal was calculated as the mean of all evaluated fields. This method is widely used in BLM-induced murine pulmonary fibrosis models and reflects both the extent and severity of fibrotic remodelling [16].

### 2.5. Statistical Analysis

Statistical analyses were performed using IBM SPSS Statistics for Windows, Version 25.0 (IBM Corp., Armonk, NY, USA). Continuous variables were expressed as mean ± standard deviation or median (interquartile range) depending on the distribution of the data. Group comparisons were performed using Student’s *t*-test or the Mann–Whitney U test as appropriate. Categorical variables were compared using the chi-square test or Fisher’s exact test. Interobserver agreement for LUS findings was assessed using Cohen’s kappa coefficient with corresponding 95% confidence intervals. Intraobserver variability was not evaluated because each observer performed a single independent assessment of the ultrasound images. Correlations between LUS scores and Ashcroft histopathological scores were analyzed using Spearman’s rank correlation coefficient. Where multiple comparisons were performed, Bonferroni’s correction was applied. A two-tailed *p* value < 0.05 was considered statistically significant.

## 3. Results

### 3.1. Lung Ultrasound Findings

**Figure 1:**
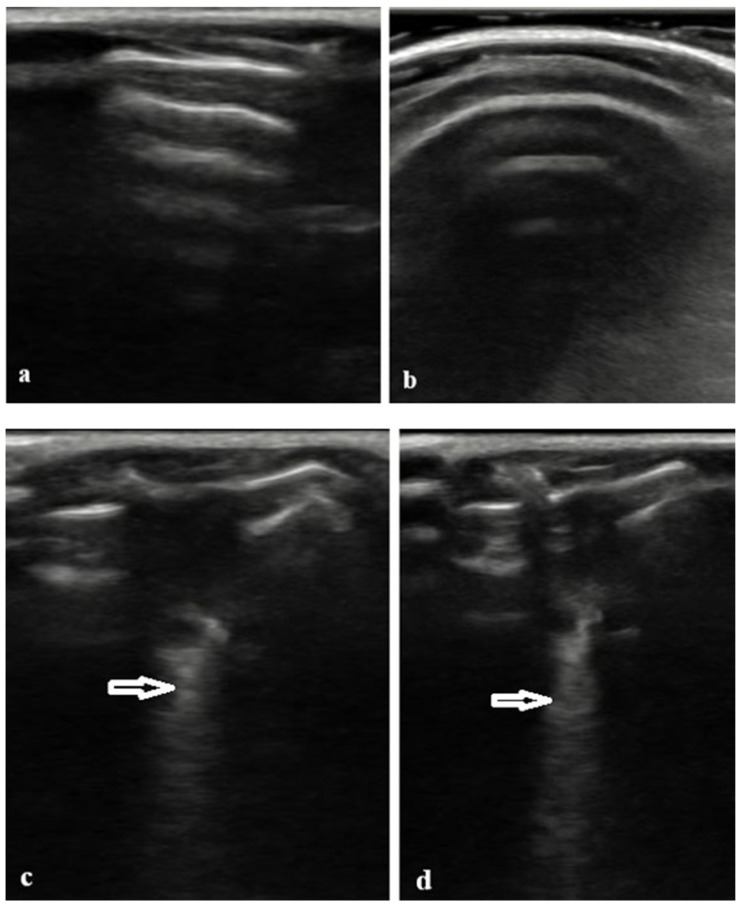
Representative LUS findings in the control group and BLM-treated group. (**a**,**b**) Normal LUS appearance in the control group: Please note the presence of prominent A-lines are visible as horizontal reverberation artifacts, indicating normally aerated lung parenchyma; these findings were consistently observed in all control mice. (**c**,**d**) LUS appearance in the BLM-treated group: Please note the presence of multiple B-lines, defined as vertical, hyperechoic artifacts arising from the pleural line and extending to the bottom of the screen without fading (arrows); these are indicative of interstitial involvement or fibrosis.

In the control group, LUS consistently demonstrated normal aeration patterns characterized by horizontal A-lines and smooth, continuous pleural lines. No pathological B-line artefacts were detected by either observer (radiologist or rheumatologist) in any control animal (Table 1). Representative normal LUS images illustrating preserved A-line patterns in control mice are shown in Figure 1a,b.

In contrast, LUS examinations in the BLM group revealed findings consistent with interstitial lung involvement. These abnormalities included the presence of multiple vertical B-lines, partial or complete loss of normal A-line patterns, and irregular or thickened pleural lines. Representative examples of B-line artifacts observed in BLM-treated mice are shown in Figure 1c,d. Both observers recorded higher LUS scores in the BLM group compared with controls.

**Figure 2:**
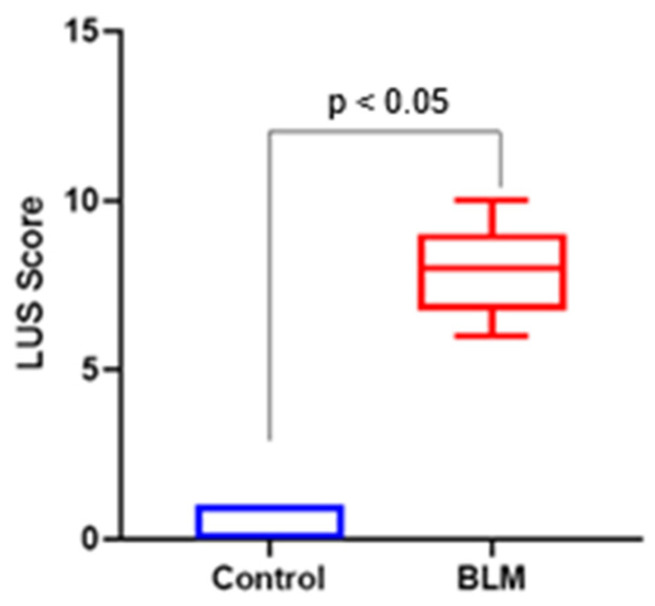
Comparison of lung ultrasound (LUS) B-line scores between control and bleomycin (BLM)-treated mice at day 21. B-lines were scored semi-quantitatively in predefined thoracic regions (0–3 per region) according to their number and degree of confluence, and total LUS scores were calculated by summing regional scores. Data are presented as median with interquartile range. Statistical comparisons between groups were performed using the Mann–Whitney U test. *p* < 0.05 versus control.

Quantitatively, median LUS scores assessed by the radiologist (LUS-1) were significantly higher in the BLM group than in the control group (median: 2.5, IQR: 2–3 vs. 0, IQR: 0–0; *p* < 0.001). Similarly, LUS scores assessed by the rheumatologist (LUS-2) were also significantly elevated in the BLM group compared with controls (median: 1, IQR: 0–1 vs. 0, IQR: 0–0; *p* < 0.001) (Figure 2).

### 3.2. Histopathological Findings and Ashcroft Scores

**Figure 3:**
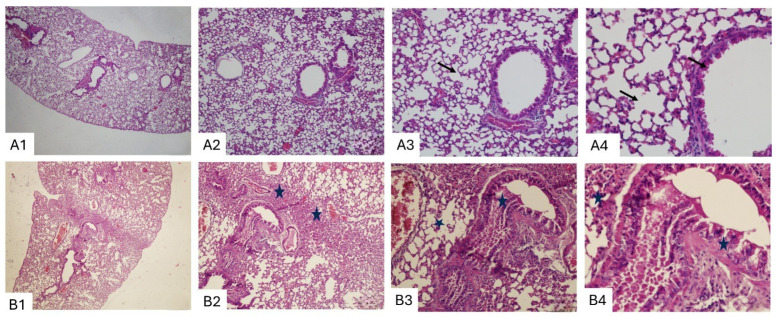
Representative lung sections stained with hematoxylin and eosin (H&E) from control (**A**) and bleomycin (BLM)-treated (**B**) mice at ×4, ×10, ×20, and ×40 magnifications. Control lungs demonstrate preserved alveolar architecture with thin (arrow) septa and normal terminal bronchioles (**A1**–**A4**). In contrast, BLM-treated lungs show marked fibrotic changes, including bronchiolar distortion (star), inflammatory cell infiltration, and thickening of alveolar septa (**B1**–**B4**).

**Figure 4:**
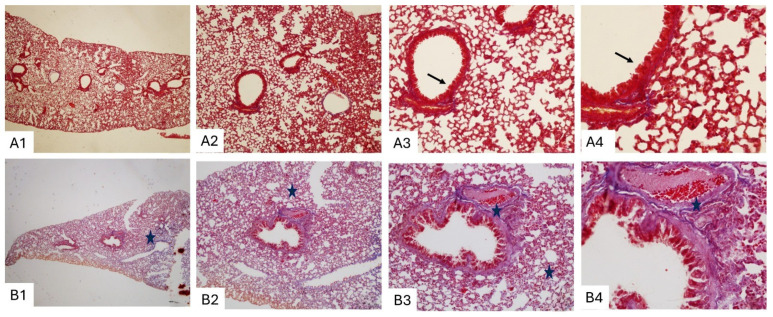
Representative lung sections stained with Masson’s trichrome from control (**A**) and bleomycin (BLM)-treated (**B**) mice at ×4, ×10, ×20, and ×40 magnifications. Control lungs show minimal collagen deposition with preserved alveolar architecture (**A1**–**A4**). In contrast, BLM-treated lungs exhibit dense collagen accumulation around terminal bronchioles (star) and within the interalveolar septa (arrows), consistent with pulmonary fibrosis (**B1**–**B4**).

Representative histological sections of lung tissue from control and BLM-treated mice are shown in Figure 3 and Figure 4. In the control group, hematoxylin and eosin (H&E)–stained sections demonstrated preserved lung architecture across all magnifications (×4, ×10, ×20, and ×40). Alveolar septa were thin and intact, terminal bronchioles showed normal morphology, and no evidence of inflammatory cell infiltration, septal thickening, or fibrotic remodelling was observed (Figure 3A1–A4).

In contrast, lung sections from BLM-treated mice revealed pronounced histopathological alterations consistent with pulmonary fibrosis. These changes included marked thickening of alveolar septa, distortion of normal alveolar architecture, inflammatory cell infiltration, and prominent bronchiolar fibrotic changes (Figure 3B1–B4). In several regions, partial to complete obliteration of alveolar spaces was evident.

Masson’s trichrome staining further highlighted the extent of fibrotic remodeling. Control lungs showed minimal collagen deposition limited to peribronchial and vascular regions, with preserved alveolar architecture (Figure 4A1–A4). In contrast, BLM-treated lungs exhibited dense collagen accumulation around terminal bronchioles and within the interalveolar septa, confirming extensive interstitial fibrosis (Figure 4B1–B4).

**Figure 5:**
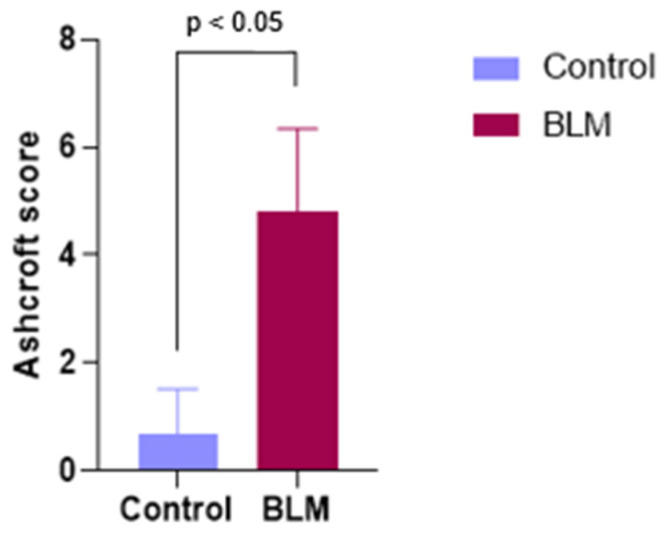
Histological assessment of pulmonary fibrosis using the Ashcroft scoring system in control and bleomycin (BLM)-treated mice at day 21. Lung sections stained with hematoxylin and eosin (H&E) were evaluated in a blinded manner, and fibrosis severity was graded on a scale from 0 (normal lung architecture) to 8 (total fibrotic obliteration). Data are presented as median with interquartile range. Statistical comparisons between groups were performed using the Mann–Whitney U test. *p* < 0.05 versus control.

Quantitative assessment using the Ashcroft scoring system corroborated these qualitative findings. The control group exhibited minimal fibrosis (median Ashcroft score: 1, interquartile range [IQR]: 0–1), whereas the BLM group demonstrated moderate to severe fibrosis (median Ashcroft score: 5, IQR: 4–6). This difference was statistically significant (Mann–Whitney U test, *p* < 0.001) (Figure 5).

### 3.3. Correlation Between Lung Ultrasound and Histopathology

In BLM-treated animals, a strong positive correlation was observed between LUS scores assessed by the radiologist (LUS-1) and histological fibrosis severity quantified using the Ashcroft scoring system (Spearman’s ρ = 0.78, *p* < 0.001). LUS scores assessed by the rheumatologist (LUS-2) also demonstrated a positive correlation with Ashcroft scores, although of lower magnitude (Spearman’s ρ ≈ 0.62, *p* < 0.01).

These findings indicate that increasing B-line burden detected by LUS reflects greater histological fibrosis severity, with stronger concordance observed for the radiologist’s assessments. Individual Ashcroft scores and corresponding LUS scores for both observers are summarized in Table 2.

Together with histopathological findings (Figure 3, Figure 4 and Figure 5), these results support the ability of LUS to semiquantitatively reflect pulmonary fibrosis severity in this experimental model, while also highlighting moderate interobserver variability.

### 3.4. Interobserver Variability

Comparison of LUS scores between the radiologist and the rheumatologist demonstrated moderate interobserver agreement. When LUS findings were dichotomised as fibrosis present (LUS score ≥ 1) or absent (LUS score = 0), the interobserver agreement was moderate, consistent with Cohen’s kappa values in the moderate agreement range. Discrepancies between observers were more pronounced in animals with intermediate Ashcroft scores, whereas agreement was high in animals with either no fibrosis or advanced fibrosis.

## 4. Discussion

This study demonstrated that LUS can identify interstitial changes associated with pulmonary fibrosis in a BLM-induced mouse model of SSc. Although histopathological evaluation confirmed pulmonary fibrosis in all BLM-treated mice, the extent to which fibrotic involvement was detected by LUS varied between observers, resulting in moderate interobserver agreement. Importantly, quantitative analysis revealed a strong correlation between LUS scores and histological fibrosis severity, supporting the ability of LUS to reflect disease burden rather than merely detect its presence.

LUS has increasingly been utilized in the bedside evaluation of a wide spectrum of pulmonary conditions, including pneumonia, pulmonary edema, pleural effusion, and ILDs [17,18]. Its non-invasive nature, real-time imaging capability, portability, and lack of ionizing radiation make it particularly advantageous for repeated assessments in both clinical and experimental settings [19]. In lung ultrasound, A-lines represent horizontal reverberation artifacts indicating normally aerated lung, whereas B-lines are vertical, hyperechoic artifacts arising from the pleural line and extending to the bottom of the screen without fading. The presence of multiple B-lines reflects increased lung density due to thickening of interstitial or alveolar structures, as observed in pulmonary fibrosis, edema, or inflammatory processes [20].

In the context of ILD, B-lines are considered sensitive—although not disease-specific—markers of subpleural interstitial involvement. Several clinical studies have demonstrated that LUS can detect interstitial lung involvement in patients with SSc, with moderate to strong correlations reported between B-line burden, HRCT findings, and pulmonary function parameters [21]. A recent systematic review reported that LUS sensitivity for detecting SSc-associated ILD ranged from 74% to 100%, although specificity varied widely (16–99%), reflecting heterogeneity in study designs and diagnostic thresholds [22]. Similarly, Gutierrez et al. demonstrated that lung ultrasound could detect subclinical ILD in approximately 59% of asymptomatic SSc patients, achieving a sensitivity of 91.2% and a specificity of 88.6% [23].

Our findings are consistent with these clinical observations and we have extended them to an experimental SSc model. In the present study, control animals consistently demonstrated normal LUS patterns with preserved A-lines and absence of pathological B-lines, and no false-positive findings were observed by either observer. In contrast, BLM-treated mice exhibited multiple B-lines, pleural line irregularities, and loss of normal aeration patterns. Moreover, LUS scores—particularly those assessed by the radiologist—showed a strong positive correlation with Ashcroft histological scores, indicating that increasing B-line burden reflects increasing fibrotic severity [22,24]. This strengthens the biological plausibility of LUS as a semiquantitative imaging tool in experimental pulmonary fibrosis.

BLM is a chemotherapeutic agent well known for its dose-dependent pulmonary toxicity and is widely used to induce experimental lung fibrosis. BLM causes direct alveolar epithelial injury, followed by inflammatory cell recruitment, fibroblast activation, and excessive collagen deposition, ultimately resulting in fibrotic remodeling of lung tissue [11,25,26,27]. In animal studies, BLM-induced ILD models have been employed to evaluate fibrosis severity using a variety of imaging approaches, including ultrasonographic grading and texture-based analysis. Zhou et al. [11], for example, demonstrated that ex vivo ultrasound parameters such as surface smoothness, echo density, and lesion margin angle differed significantly between control, mildly fibrotic, and severely fibrotic mouse lungs [28].

Histopathological confirmation of fibrosis in all BLM-treated animals in our study supports the robustness and reproducibility of the experimental model and confirms the fibrogenic effect of subcutaneous BLM administration [12,14]. The absence of fibrosis in the control group further validates model specificity. Masson’s trichrome staining provided clear visualization of collagen accumulation and architectural distortion, allowing reliable grading of fibrosis severity using the Ashcroft scoring system. Importantly, the correlation observed between Ashcroft scores and LUS findings indicates that LUS captures clinically relevant structural alterations rather than incidental artifacts.

Interobserver variability represents a critical aspect of ultrasound-based assessments. In the present study, agreement between the radiologist and the rheumatologist was moderate, with discrepancies most pronounced in animals with intermediate fibrosis severity, where ultrasonographic findings are often more subtle. This observation is consistent with previous reports emphasizing the operator-dependent nature of LUS and the importance of experience and training in ultrasound interpretation [10,28]. Both observers had approximately 10 years of ultrasound experience; however, their areas of expertise differed. The radiologist had greater experience in thoracic and pulmonary ultrasound interpretation, whereas the rheumatologist primarily used ultrasound in musculoskeletal and rheumatologic imaging. This difference in domain-specific expertise may partly explain the stronger correlation observed between radiologist-assessed LUS scores and histological fibrosis severity.

Interobserver agreement for LUS findings was moderate (κ = 0.47). Agreement was highest in animals with either minimal or advanced fibrosis, whereas discrepancies were mainly observed in those with intermediate disease severity, where ultrasonographic findings are more subtle. The stronger correlation between radiologist-assessed LUS scores and Ashcroft scores compared with rheumatologist-assessed scores may reflect differences in experience with thoracic ultrasound interpretation. Because the LUS scoring system is ordinal, alternative agreement metrics such as weighted kappa or intraclass correlation coefficients could also be considered in future studies to better capture gradations of disease severity [18].

To improve reproducibility, LUS examinations in the present study were performed using a standardized imaging protocol, including a high-frequency linear probe, fixed acquisition parameters, predefined thoracic scanning zones, and semi-quantitative B-line scoring based on international recommendations. Observers were blinded to group allocation and analyzed images independently. Nevertheless, further standardization across ultrasound systems, operator training, and the development of automated or quantitative image analysis tools will be necessary to enhance reliability and facilitate translation to clinical SSc-ILD assessment [29].

An ideal monitoring tool for ILD should be non-invasive, repeatable, radiation-free, and capable of detecting changes in lung involvement over time. LUS fulfills many of these criteria, as it allows bedside assessment, does not expose subjects to ionizing radiation, and can be repeated frequently without procedural risk. These characteristics make LUS particularly attractive for longitudinal monitoring in both experimental models and clinical ILD settings [10,30].

The present findings may also have important translational implications. Although this study was conducted in a controlled murine model of pulmonary fibrosis, the observed association between LUS findings and histopathological fibrosis severity suggests that LUS may reflect underlying structural lung alterations. Future studies are needed to investigate whether standardized LUS scoring systems correlate with HRCT, pulmonary function parameters, and disease activity in patients with SSc–associated ILD. Longitudinal clinical studies may further clarify the potential role of LUS in disease monitoring and treatment response assessment [31,32].

From a translational perspective, these findings suggest that LUS may be best positioned as a complementary and longitudinal monitoring tool rather than a standalone surrogate for histology in experimental ILD models. Its strengths lie in its non-invasive nature and feasibility for repeated measurements, whereas its limitations include operator dependency and reduced sensitivity for detecting early or mild fibrotic changes confined to deeper lung regions [10,18,28]. The moderate interobserver variability observed in this study highlights the necessity of protocol standardization, formal training, and potentially automated or quantitative image analysis techniques to enhance reproducibility.

Several limitations should be acknowledged. First, the relatively small sample size, dictated by ethical considerations and the 3R principles of animal research, may limit the generalizability of the findings. Future studies including larger cohorts are warranted to confirm reproducibility and refine the diagnostic performance of LUS in experimental pulmonary fibrosis models. Second, intra-observer variability and measurement stability across different ultrasound systems, probes, and scanning angles were not evaluated in this study. Although standardized imaging conditions were used, future studies including repeated measurements and multi-device validation are required to determine the reproducibility of LUS findings. Third, LUS assessments were performed at a single time point during the fibrotic phase, precluding evaluation of temporal disease progression. Fourth, imaging was conducted at a single time point corresponding to the fibrotic phase of the model, precluding assessment of disease evolution. Longitudinal studies with serial ultrasonography and histopathological correlation are needed to clarify the temporal dynamics of fibrosis and the utility of LUS for monitoring progression or treatment response. Fifth, although Ashcroft scoring provides a widely accepted semiquantitative assessment of fibrosis, future studies incorporating objective collagen quantification or three-dimensional imaging may further strengthen structure–function correlations. Sixth, intraobserver variability was not assessed in this study, as each observer evaluated the images only once. Therefore, the stability of measurements over repeated assessments could not be determined and should be investigated in future studies. Finally, anesthesia-related effects on lung aeration were not formally controlled or quantified, and anesthetic-induced atelectasis may have influenced ultrasound findings.

## 5. Conclusions

LUS detected pulmonary abnormalities and demonstrated a significant association with histological fibrosis severity in this experimental model. However, moderate interobserver variability and the limited sample size warrant cautious interpretation. These findings suggest that LUS may serve as a useful non-invasive adjunct for the semiquantitative assessment of pulmonary fibrosis in experimental settings.

This study demonstrates that LUS can detect pulmonary involvement and provide a semiquantitative estimate of fibrosis severity in a BLM-induced SSc mouse model, showing significant correlation with histopathological findings. Moderate interobserver variability—particularly in intermediate disease stages—highlights the importance of standardized imaging protocols and operator expertise. Overall, LUS may represent a valuable non-invasive adjunctive imaging modality for experimental pulmonary fibrosis research; however, its ability to reliably estimate fibrosis severity requires confirmation in larger cohorts and longitudinal studies.

## Figures and Tables

**Table 1:** Histological Ashcroft Scores and Lung Ultrasound Findings in the Control Group. Histological fibrosis severity, assessed by the Ashcroft score, and LUS findings were evaluated by a radiologist (LUS-1) and a rheumatologist (LUS-2) in the control group. No pathological B-line artifacts were detected in the majority of the control.

Mouse ID	Ashcroft Score	LUS—1 (Radiologist)	LUS—2 (Rheumatologist)
M1	0	0	0
M2	0	0	0
M3	1	0	0
M4	2	1	1
M5	1	0	0
M6	0	0	0
M7	2	1	1
M8	0	0	0
M9	1	1	0
M10	0	0	0

**Table 2:** Histological Ashcroft Scores and Lung Ultrasound Findings in the Bleomycin Group. Relationship between histological fibrosis severity (Ashcroft score) and LUS findings assessed by two independent observers in bleomycin-treated mice. Higher LUS scores, particularly those assessed by the radiologist, were associated with increased fibrosis severity.

Mouse ID	Ashcroft Score	LUS—1 (Radiologist)	LUS—2 (Rheumatologist)
M1	7	3	3
M2	6	3	1
M3	6	3	0
M4	3	1	0
M5	4	2	0
M6	5	2	1
M7	2	0	0
M8	4	2	2
M9	5	3	1
M10	6	3	0

## Data Availability

The data that support the findings of this study are available from the corresponding author upon reasonable request.

## Abbreviations

The following abbreviations are used in this manuscript:

SSc	Systemic sclerosis
LUS	Lung ultrasonography
BLM	Bleomycin
ILD	Interstitial lung disease
HRCT	High-resolution computed tomography
